# SpliceSeq: a resource for analysis and visualization of RNA-Seq data on alternative splicing and its functional impacts

**DOI:** 10.1093/bioinformatics/bts452

**Published:** 2012-07-20

**Authors:** Michael C. Ryan, James Cleland, RyangGuk Kim, Wing Chung Wong, John N. Weinstein

**Affiliations:** ^1^In Silico Solutions, Fairfax, VA and ^2^Department of Bioinformatics and Computational Biology and Department of Systems Biology, The University of Texas MD Anderson Cancer Center, Houston, TX, USA

## Abstract

**Summary:** SpliceSeq is a resource for RNA-Seq data that provides a clear view of alternative splicing and identifies potential functional changes that result from splice variation. It displays intuitive visualizations and prioritized lists of results that highlight splicing events and their biological consequences. SpliceSeq unambiguously aligns reads to gene splice graphs, facilitating accurate analysis of large, complex transcript variants that cannot be adequately represented in other formats.

**Availability and implementation:** SpliceSeq is freely available at http://bioinformatics.mdanderson.org/main/SpliceSeq:Overview. The application is a Java program that can be launched via a browser or installed locally. Local installation requires MySQL and Bowtie.

**Contact:** mryan@insilico.us.com

**Supplementary Information:** Supplementary data are available at *Bioinformatics* online.

## 1 INTRODUCTION

The majority of human genes can express alternative transcripts as a function of tissue type, developmental stage or physiological context, and aberrant splicing patterns can lead to disease, notably cancer. Nonetheless, most analysis pipelines remain tied to a one-gene, one-protein, one-function model despite the incisive, high-resolution data now provided by next-generation sequencing.

Our web-based SpliceCenter resource (Kahn *et al.* 2007; Ryan *et al.*, 2008) provides the laboratory biologist with a user-friendly resource for visually analyzing the impact of splice variation on such technologies as reverse transcription–polymerase chain reaction (RT–PCR), microarrays, RNAi and peptide agents. However, it does not provide tools for detailed analysis and visualization of the rich, new data from RNA-Seq. Hence, we have developed SpliceSeq to fill that gap.

Several other analytical approaches have been used to analyze the relative abundance of various transcript isoforms in a sample using the short reads provided by RNA-Seq. See, e.g. Katz *et al.* (2010) and Trapnell *et al.* (2012). Those methods have generally predicted isoform expression levels by making probabilistic assignments of reads to matching isoforms, paying particular attention to reads that cross splice junctions. Some also include inferences from paired-end insert length. Those approaches have delivered ground-breaking results, but there are limitations. For example, many RNA-Seq analysis tools lack detailed protein annotations that can identify functional impacts of alternative splicing. Most serious, they do not readily handle genes with many densely distributed alternative splice paths or splice events separated by more than the read length (or insert length of paired-end reads). Those difficult cases seemed rare when we originally explored transcript databases such as RefSeq, but more comprehensive databases now show complex splicing patterns for the majority of multi-exon genes. In our own database of alternative splice forms, ∼54% of human genes have multiple alternative splice events separated by >750 bases, the average paired-end insert length.

Instead of probabilistically assigning reads across isoforms, we base our analysis on splice graphs. Splice graphs with exons as nodes and splices as edges have been used previously in transcriptome analysis for assembly of ESTs and short reads (Xing *et al.*, 2004). We use them here because short reads can be mapped unambiguously to the graph, providing an intuitive, composite view of alternative splicing. Furthermore, recursive graph traversal weighted by depth of read provides a prediction of the protein sequences produced by the isoforms, and UniProt annotations indicate functional elements impacted by the splicing. Our initial implementation of SpliceSeq includes only human genes and is designed to detect only known exons and splices. However, the algorithms are equally applicable to other species and can be extended to detect novel splice events. Those extensions are in development.

## 2 METHODS AND IMPLEMENTATION

SpliceSeq is a Java application for visualization and quantitation of RNA-Seq reads and their potential functional consequences in the context of transcript splice graphs. It can be launched from a web browser through Java Web Start or downloaded for local installation. The Web Start version allows the user to analyze preloaded RNA-Seq data. The local version allows users to process their own samples. A MySQL database and the Bowtie sequence aligner (Langmead *et al.*, 2009) are required for local installation.

SpliceSeq aligns reads to a pre-constructed set of gene models (splice graphs, one per gene) assembled from a reference collection of splice variants. We currently use Ensembl transcripts to construct our graphs but can use transcripts from a variety of sources. Each gene is represented by a single graph with exons as nodes and splices as edges. Each portion of the gene's sequence maps to a single unique location in the graph.

When RNA-Seq data are imported, a bowtie database is generated for alignment of the reads to the splice graphs. The alignment database consists of overlapping tiles generated by recursive traversal of each splice graph. Tile length is optimized based on the read length of the RNA-Seq data (or insert length for paired end reads).

Using Bowtie and the alignment database, SpliceSeq aligns reads to the graph, keeping totals for the number of times an exon or splice junction is observed. Raw totals and what we term ‘OPKM’ (observation totals normalized by exon length and million-aligned-reads) are generated. In calculating RPKM (reads per kilobase of transcript per million aligned reads), a given read is counted just once. In contrast, if a read crosses two splice junctions and three exons, each of those graph elements is ‘observed’ by the read and contributes to the calculated value of OPKM.

After reads have been aligned to the splice graphs, two forms of analysis are performed. First, the patterns of observed transcript splicing are evaluated for alternative splicing events. Events are categorized by type (e.g. exon skip, cassette exons and alternate promoter). Second, a weighted traversal of each gene's splice graph is performed to predict resulting alternative protein sequences. UniProt annotations are then inspected to identify protein changes that impact known functional elements.

SpliceSeq has options for analysis of individual samples and for comparison of matched samples. For individual samples, it identifies genes with multiple splice forms, classifies splice events and shows functional elements altered by splicing. Splice junction reads are the basis for that analysis, and exon reads are used to confirm the classification of events. In accord with criteria suggested previously (Wang *et al.*, 2008), for a splice junction to be considered observed, it must have two or more reads that overlap into each exon by ≥4 bases. In the comparison option, SpliceSeq identifies genes with splicing differences between the samples, classifies the events and predicts biological impact. To compare splice junction and exon read totals between samples, it calculates a splice index (SI) value:

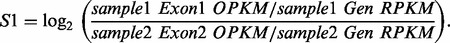



The SI value identifies changes in splicing that are distinct from changes in gene expression. A *P*-value for each splice/exon is also calculated using a Fisher's exact test on a 2 × 2 contingency table (see Supplemental Methods).

## 3 RESULTS

SpliceSeq provides an interactive interface focused on exploration of alternative transcripts with the goal of elucidating splicing changes that impact function. For either a single sample or comparison of samples, a spreadsheet-style table of genes is presented. The list can be searched, sorted, filtered or exported in order to zero in on genes of interest, types of splice events or impacted UniProt features (e.g. domains). Above the gene list is a graphical display that shows the transcript-level splice graph or the protein form(s) translated from the selected gene. In the graphical display, color and numerical annotations identify alternative splicing events. The graph can show OPKM, raw read totals or SI values for comparisons. The protein pane (see Supplementary Fig. S1) presents protein sequences that result from traversal of the splice graph, weighted by observed read depth. The protein pane predicts the relative ratio of alternate protein species and displays the functional elements contained in each.

## 4 DISCUSSION

The importance of alternative messenger RNA (mRNA) splicing to biological function is sometimes unclear. For example, it is not yet known how many of the frequent splicing changes seen in cancer are central to carcinogenesis and how many are a byproduct of it. To address such questions, SpliceSeq uses splice graphs for clear, intuitive identification and visualization of differences in splicing that affect function. The splice graph analysis avoids the difficult challenge of disambiguating RNA-Seq reads in an increasingly complex isoform landscape. The splice graph visualization simply and accurately presents the expression levels of transcript elements, supporting identification of known functional elements impacted by alternative splicing. RNA-Seq data can be visualized in a genome browser, but SpliceSeq has the advantages that it removes intron space, statistically summarizes exon/splice expression, adds functional annotations and supports sample comparison.

We have validated SpliceSeq's isoform prediction by simulation testing in comparison with the widely used program, Cufflinks (Trapnell *et al.*, 2012). Both algorithms performed well for most isoforms, but SpliceSeq had a slight edge in terms of the correspondence between computed and actual splice form expression in the simulations (On average, SpliceSeq was within 13% of actual concentration versus 15% for Cufflinks—see Supplementary document on Simulation Testing for details). SpliceSeq performed particularly well on the quantitation of low-frequency isoforms. That may be particularly advantageous for application to tumors, which are heterogeneous in non-standard ways. The strongest differentiation between the two programs, however, is that SpliceSeq provides user-friendly, interactive graphics and isoform-specific functional annotations.

**Fig. 1.**
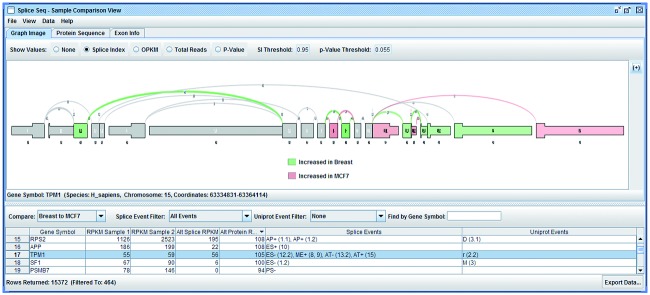
SpliceSeq comparison view showing complex splicing changes in the tumor suppressor gene TPM1 in normal breast tissue versus an MCF7 breast cancer cell line

*Funding*: Supported in part by Grant #U24CA143883 from the U.S. National Cancer Institute (UT-MD Anderson TCGA Genome Data Analysis Center), by a grant from the Michael and Susan Dell Foundation (honoring Lorraine Dell) and by generous gifts from the H.A. and Mary K. Chapman Foundation and an anonymous donor.

*Conflict of Interest*: none declared.
